# Climate oscillations, glacial refugia, and dispersal ability: factors influencing the genetic structure of the least salmonfly, *Pteronarcella badia* (Plecoptera), in Western North America

**DOI:** 10.1186/s12862-015-0553-4

**Published:** 2015-12-12

**Authors:** John S. Sproul, Derek D. Houston, C. Riley Nelson, R. Paul Evans, Keith A. Crandall, Dennis K. Shiozawa

**Affiliations:** Department of Integrative Biology, Oregon State University, 3029 Cordley Hall, Corvallis, OR 97333 USA; Department of Ecology, Evolution and Organismal Biology, 251 Bessey Hall, Ames, IA 50011 USA; Department of Biology, Brigham Young University, 4102 Life Science Building, Provo, UT 84602 USA; M.L Bean Life Science Museum, Brigham Young University, 645 East 1430 North, Provo, UT 84602 USA; Department of Microbiology and Molecular Biology, Brigham Young University, 4007 Life Science Building, Provo, UT 84602 USA; Computational Biology Institute, George Washington University, Innovation Hall, Ashburn, VA 20147 USA

**Keywords:** Stoneflies, Next-generation sequencing, Phylogeography, Last Glacial Maximum, Cryptic genetic diversity

## Abstract

**Background:**

Phylogeographic studies of aquatic insects provide valuable insights into mechanisms that shape the genetic structure of communities, yet studies that include broad geographic areas are uncommon for this group. We conducted a broad scale phylogeographic analysis of the least salmonfly Pteronarcella badia (Plecoptera) across western North America. We tested hypotheses related to mode of dispersal and the influence of historic climate oscillations on population genetic structure. In order to generate a larger mitochondrial data set, we used 454 sequencing to reconstruct the complete mitochondrial genome in the early stages of the project.

**Results:**

Our analysis revealed high levels of population structure with several deeply divergent clades present across the sample area. Evidence from five mitochondrial genes and one nuclear locus identified a potentially cryptic lineage in the Pacific Northwest. Gene flow estimates and geographic clade distributions suggest that overland flight during the winged adult stage is an important dispersal mechanism for this taxon. We found evidence of multiple glacial refugia across the species distribution and signs of secondary contact within and among major clades.

**Conclusions:**

This study provides a basis for future studies of aquatic insect phylogeography at the inter-basin scale in western North America. Our findings add to an understanding of the role of historical climate isolations in shaping assemblages of aquatic insects in this region. We identified several geographic areas that may have historical importance for other aquatic organisms with similar distributions and dispersal strategies as *P. badia*. This work adds to the ever-growing list of studies that highlight the potential of next-generation DNA sequencing in a phylogenetic context to improve molecular data sets from understudied groups.

**Electronic supplementary material:**

The online version of this article (doi:10.1186/s12862-015-0553-4) contains supplementary material, which is available to authorized users.

## Background

Molecular studies in a phylogeographic context provide insights into the evolutionary history of the taxon of interest, and as studies across taxa accumulate, inference of broader deterministic processes is possible. Such studies are particularly valuable in understanding factors that impact complex, diverse communities as seen in freshwater aquatic systems. Insects account for much of the diversity present in freshwater communities [1]. With a wide array of dispersal abilities and habitat tolerance [2], aquatic insects provide researchers a host of candidate taxa for testing phylogeographic hypotheses at many scales [3].

While aquatic insects have been reasonably well studied for many decades, relatively few molecular studies are conducted given the number of aquatic taxa. Studies that do exist are often limited in geographic scale [4]. Yet studies that have considered large geographic areas have provided powerful insights on the effect of historical climatic processes on genetic structure at the species and community level [5–9], as well as the importance of long-distance dispersal [10].

A significant obstacle to conducting molecular studies in aquatic insects is the lack of genomic information for many taxa of interest. Until recently, researchers have been confined to using markers available through universal or degenerate primers such as the “barcode” region of the mitochondrial DNA (mtDNA) cytochrome oxidase I (CO1) gene [11, 12]. For phylogenetic purposes, this may not be sufficient to produce a well-supported gene tree. The ability to generate large amounts of genomic data at ever decreasing costs through next-generation sequencing approaches makes it feasible for investigators to move past constraints on genomic information in the early stages of a research project; thus, enabling them to conduct more effective molecular studies in non-model groups.

Here we present a phylogeographic study of the least salmonfly *Pteronarcella badia*. This herbivorous stonefly is moderately sized and occurs in mid-elevation mountain streams across western North America. It is one of two members of the genus *Pteronarcella* within the family Pteronarcyidae (Plecoptera). The species is readily identifiable in the field in its immature form and as an adult (except where its range overlaps with its sister species *P. regularis* (Hagen) in the Pacific Northwest, there the immature stages cannot be distinguished). When it is present it often occurs abundantly. The broad western North American distribution of *P. badia* makes it an effective organism to study phylogeographic patterns in this region, which has few studies of aquatic insects from a similar geographic scale to date (but see [10, 13]). Degenerate and barcode primers failed to amplify reliably across several sample localities in preliminary experiments with polymerase chain reaction (PCR), potentially because of mutations in primer-binding sites for this taxon. To generate mtDNA primers for COI as well as for other rapidly evolving mtDNA genes for which reliable primers were not available, we used 454 pyrosequencing to sequence the complete mitochondrial genome (mt genome) in the early stages of the research. From the mt genome, we developed PCR primer pairs to amplify three fragments spanning portions of five protein coding mtDNA genes: ATP synthase subunit 6 (ATP6), COI, cytochrome oxidase III (COIII), cytochrome *b* (CYTB), and NADH dehydrogenase 6 (ND6). We combined sequence data from these five mtDNA genes with nuclear rDNA 28S to form a dataset designed to address the following research questions: (1) *What is the population structure of P. badia?* (2) *What are the dominant modes of dispersal in P. badia?* We specifically test whether dispersal through hydrologic connectivity or overland movement (putatively through flight during the winged adult stage) appears to be more important in determining genetic structure. (3) *How have historical climate oscillations influenced population structure?* We test for evidence of multiple glacial refugia, timing of interclade divergence, and patterns of demographic history to estimate the influence of historical climate cycles on the population structure of *P. badia*.

## Results

### Mitochondrial genome reconstruction

**Fig. 1:**
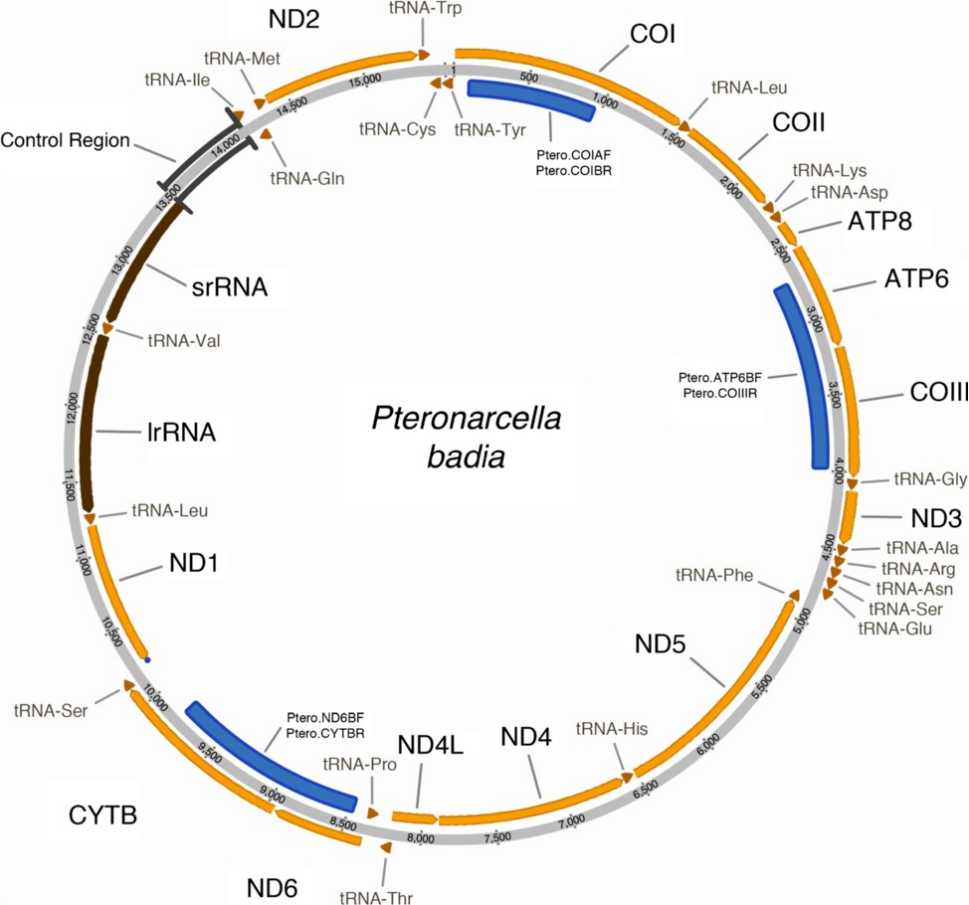
Annotated visualization of the *P. badia* mt genome. Genes located outside the gray circle occur on the majority-coding strand (J-strand), and genes inside the gray circle occur on the minority-coding strand (N-strand). Blue bars represent regions that were amplified in the present study for phylogenetic inference. Accompanying labels indicate the primer pairs used for PCR amplification (see Table 3)

Our 454 pyrosequencing produced six mtDNA contigs that represented 96.3 % (15,017 of 15,586 bp) of the total genome. PCR-based Sanger sequencing of the gaps yielded the remaining 569 bps with the majority (>500 bps) of the missing sequence coming from the A + T rich control region. Nucleotide composition showed overall A + T richness that is typical in insect mt genomes [14] with total A + T composition = 67.4 %. Sequence analysis in MOSAS identified 36 of the 37 genes expected to be present in the mt genome. Alignment with the mt genome of *Pteronarcys princeps* (GenBank #NC_006133.1) [14] confirmed the location of *tRNA*^*Arg*^, the only gene unidentified by MOSAS. No mt genome rearrangements relative to the ancestral Pancrustacean genome [15] were present as all protein-coding, rRNA, and tRNA genes occurred in the same relative genomic position as the “ancestral” genome based on comparison to *Drosophila yakuba* [16]. The complete annotated sequence is available on GenBank [17], accession #KU182360. An annotated visualization of the complete genome is shown in Fig. 1.

### Phylogenetic analysis and hypothesis testing

Following alignment and trimming, our data set consisted of 2518 bp of mitochondrial and 880 bp of nuclear DNA sequence. PCR amplification for one or more genes was unsuccessful in 13 of the 265 in-group individuals included in the analysis (mostly museum specimens with putatively degraded DNA), so they were not included in the population genetic analyses. Translation of mtDNA sequence into amino acids did not reveal any unexpected stop codons. In scanning chromatograms for ambiguous bases, we observed at least one ambiguity in 42 of the approximately 750 mitochondrial sequences generated for this study (35 CYTB/ND6 sequences, five ATP6/COIII sequences, and two COI sequences). Although a total of 119 ambiguities were observed in these 42 sequences, all but six ambiguities were due to low quality score bases positioned within 50 bases of the end amplified fragment, where calls were being made on a single (either forward, or reverse) sequence. Thus, we only found six ambiguities across all sequences for which both forward and reverse sequences showed the same ambiguous signal. Given this result, we find it unlikely that our conclusions are being appreciably influenced by the presence of pseudogenes.

#### What is the population structure?

**Fig. 2:**
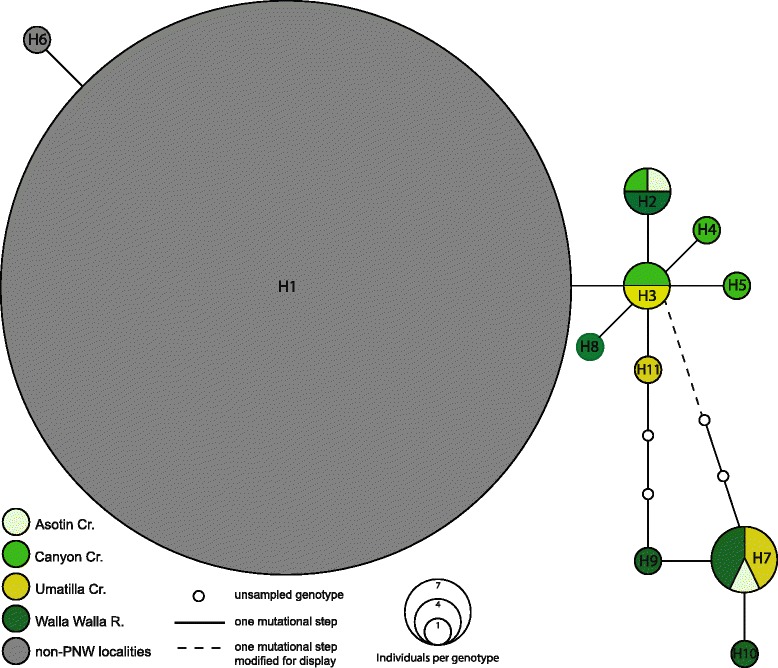
A TCS gene genealogy network of *P. badia* genotypes that was estimated using 880 bp 28S nuclear ribosomal RNA. All genotypes sampled from the Pacific Northwest clade are colored to indicate geographic location, while the genotypes of all non-Pacific Northwest localities are shown in gray

Mitochondrial DNA sequences showed high levels of variation with 383 polymorphic sites (302 = parsimony informative, 81 = singleton) and an overall nucleotide diversity of 0.025. Of the 252 individuals sampled, 151 unique haplotypes were present with an overall haplotypic diversity of 0.991 ± 0.002. TCS haplotype networks revealed very high levels of population structure with multiple sub-networks failing to connect with the connection limit set to 50 steps. High levels of population structure were also evidenced by an overall Φ_ST_ of 0.91 (*p* < 0.001). The nuclear locus showed far less variability than the mtDNA sequences, with only 15 genotypes present, and all individuals from 31 of the 38 sample localities sharing the same genotype (Fig. 2). Thirteen of the remaining genotypes were only present in six sample localities along the western edge of the species range from northern Nevada to southern Washington. The remaining genotype was from a single individual in Wyoming.

**Fig. 3:**
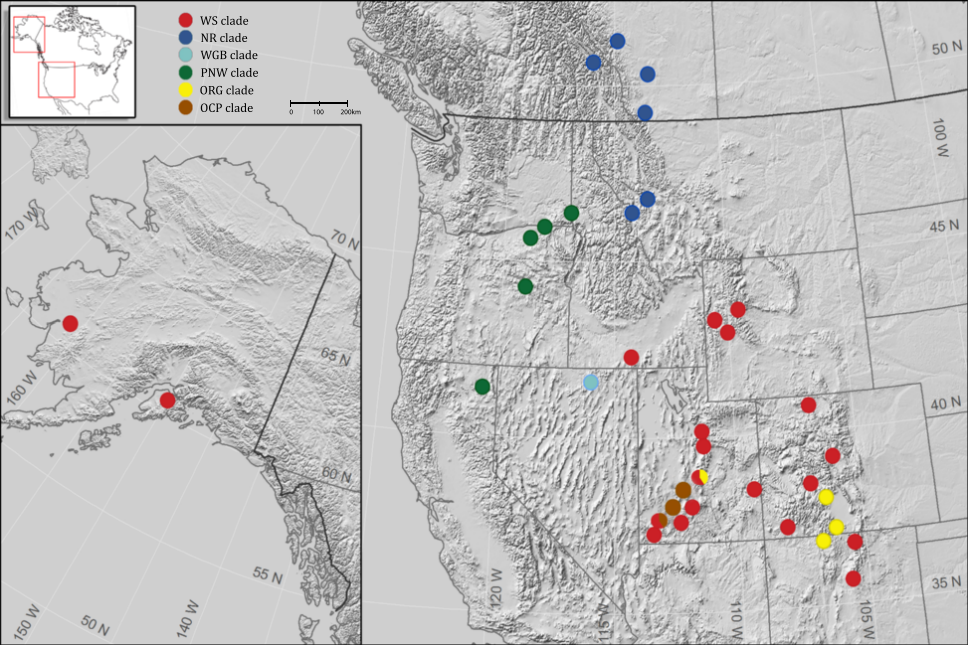
A map showing the distribution of sample localities for *P. badia* across portions western North America. Clade membership (as identified in Fig. 4) for all specimens at a given locality is represented by color. Clade names are abbreviated as follows. Widespread (WS), Northern Rockies (NR), Western Great Basin (WGB), Pacific Northwest (PNW), Old Rio Grande (ORG), and Old Colorado Plateau (OCP)

**Fig. 4:**
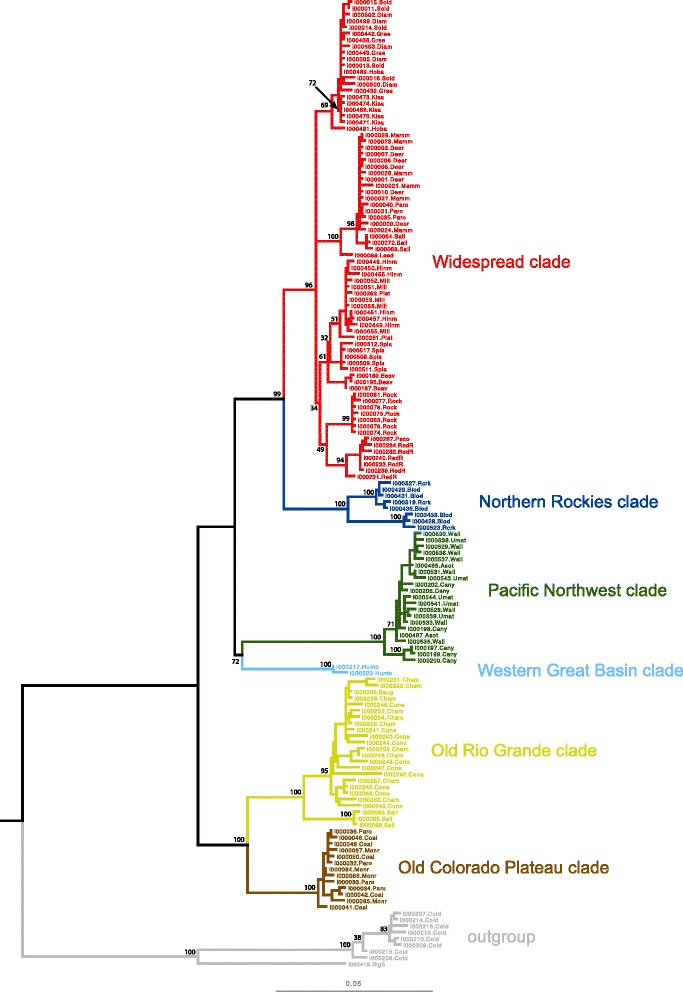
A RAxML Maximum Likelihood topology showing the relationships among *P. badia* based on a combined analysis 2518 bp of mitochondrial DNA and 880 bp of 28S nuclear ribosomal across 200 search replicates*.* Nodal support values shown at major nodes are based on a bootstrap analysis with 1000 search replicates, redundant haplotypes were omitted prior to the analysis. Each specimen name includes the BYU code followed by a suffix that abbreviates the sample locality of the specimen (BYU codes and locality suffixes are explained in Table 2). Highly divergent clades are labeled and identified by color

Tree reconstruction in RAxML produced a topology with good to excellent nodal support for all major clades (see Fig. 4). Six deeply divergent clades were present within the in-group which we labeled: Old Colorado Plateau, Old Rio Grande, Western Great Basin, Pacific Northwest, Northern Rockies, and Widespread (see Figs. 3 and 4).

The two clades that originate from the initial divergence in the *P. badia* phylogeny, the Old Rio Grande and Old Colorado Plateau clades, comprise haplotypes sampled from seven localities in the southeastern portion of the species range (central Utah, central Colorado, and northern New Mexico). Despite being highly divergent at the mitochondrial locus (up to 5.2 % pairwise distance), no changes separate these clades at the nuclear locus. The Pacific Northwest clade includes all individuals sampled from the western edge of the species’ range, from northern California to southern Washington. We excluded Thomas Creek, CA from the full data set as only COI and CYTB sequences were available for the single specimen from that locality. However, secondary analysis of COI and CYTB placed this locality in the Pacific Northwest clade. For this reason we show Thomas Creek as belonging to the Pacific Northwest clade on the distribution map shown in Fig. 3. The Pacific Northwest clade is monophyletic with the Western Great Basin clade, which contained all individuals sampled from a single locality in northern Nevada. The Pacific Northwest and Western Great Basin are the only clades supported by variation at the nuclear locus (Fig. 2).

The Northern Rockies clade includes all individuals from all localities in Montana and southern Canada. Three additional localities in Canada obtained from museum specimens (Spray River, Columbia River, and Lee Creek) were excluded from the analysis due to missing sequence data, however a separate analysis that included only sequences from COI and CYTB showed that they also fall in the Northern Rockies clade (and thus are shown in Fig. 3 as belonging to the Northern Rockies clade). The last major clade to diverge in our topology, the Widespread clade, contains haplotypes from all remaining sample localities, ranging from Alaska to New Mexico. Two sample localities (Parowan Creek and Salina Creek), both located on the Colorado Plateau, contained haplotypes from two different clades. All other sample localities only contained representatives from one of the six clades, without admixture. Near identical haplotypes (1–2 mutational steps) were present at geographically distant localities in Alaska (Kisaralik River), Wyoming (Green River) and Utah (Diamond Fork River).

Within the Widespread and Old Rio Grande clades, several moderate to well-supported subclades correlated with geographic locality were present. Within the Widespread clade, subclades include: all the haplotypes from Rock Creek (BS = 99 %), all haplotypes from the Red River and Pecos River in the southern Rocky Mountains (BS = 94 %), all haplotypes from Deer Creek, Mammoth Creek, Parowan Creek, and 3 haplotypes from Salina Creek (BS = 98 %), and all the Alaskan localities (BS = 72 %). Within the Old Rio Grande Clade, the Salina Creek haplotypes form a well-supported clade (BS = 100 %), as do all haplotypes from the Chama River, Conejos River, and Sauguache Creek (BS = 95 %).

Comparison of the total evidence tree (3398 bp) to the topology produced using only COI (745 bp after trimming) showed that while COI recovered the major clades present in the total evidence topology, relationships between clades were not identical and three of the six clades had less than 70 % bootstrap support in the COI topology.

#### Importance of hydrologic connectivity vs. overland dispersal

AMOVA results showed that 26.15 % of genetic variation was explained by differences between drainage basin (Φ_SC_ = 0.88, *p* = >0.001), differences between sample localities explained 64.87 % of the genetic variation (Φ_ST_ = 0.91, *p* = >0.001), and differences within localities explained 8.98 % of the variation (Φ_CT_ = 0.26, *p* = 0.007). Each major clade, and several subclades contained localities from multiple drainage basins (with the exception of Western Great Basin which only contained individuals from a single locality). Near identical haplotypes were observed in localities without hydrologic connections in the Widespread and Northern Rockies clades. In the Widespread clade, near identical haplotypes (1–2 mutational steps) were present in the Diamond Fork River (Colorado River drainage), the Hoback River (Columbia River drainage), and Kisaralik River (Kuskokwim drainage). In the Northern Rockies clade, near identical haplotypes (two mutational steps) were observed in Blodgett Creek (Columbia River drainage) and Lee Creek (Hudson River drainage).

**Fig. 5:**
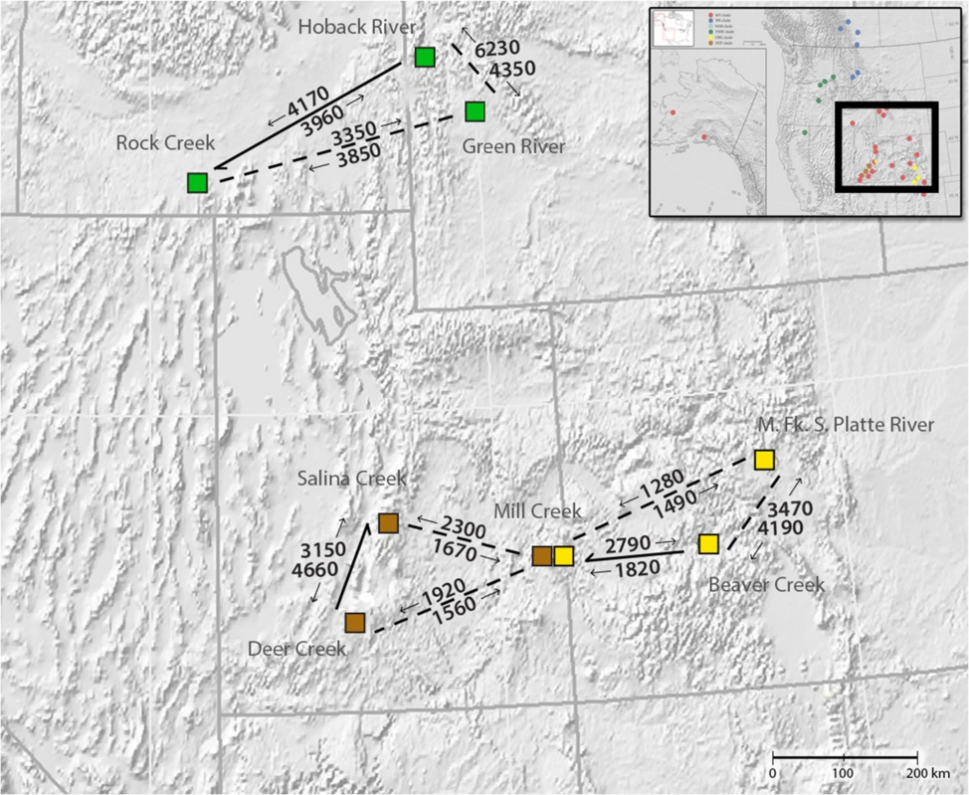
A graphical summary showing the results of three MIGRATE-n gene flow analyses. Each analysis includes three sample localities represented by green squares (Analysis 1), yellow squares (Analysis 2), and brown squares (Analysis 3). Pairwise gene flow estimates (M) are provided between each locality (M = effective migrants per generation scaled by mutation rate). Localities containing direct hydrologic connection within the same drainage basin are connected by solid lines with the direction of river flow indicated by half arrows, while localities in adjacent drainage basins are connected by dotted lines. Arrows indicate direction of gene flow for each estimate. An inset of Fig. 3 is provided in the upper right corner with the geographic area of the present figure outlined in red

Estimates of gene flow suggested that migration rates between localities in adjacent drainage boundaries may be comparable to, or higher than migration rates between geographically proximate localities with a direct hydrologic connection. The gene flow analysis is summarized in Table 1 and presented graphically in Fig. 5. Mantel tests for isolation by distance were not significant for the Old Colorado Plateau (*p* = 0.33), Old Rio Grande (*p* = 0.16), Pacific Northwest (*p* = 0.46), and Northern Rockies (*p* = 0.25) clades. Isolation by distance was significant for the Widespread clade when the Alaskan samples were included (*p* = 0.0015), and also when they were excluded (*p* = <0.001).

**Table 1:** Summary of the MIGRATE-n gene flow estimates (M) for *P. badia*

	2.50 %	0.975	Mode	Mean
ΘRock Creek (1)	0.00000	0.05967	0.00217	0.03472
ΘHoback River (2)	0.00000	0.0042	0.00103	0.04682
ΘGreen River (3)	0.00000	0.01227	0.00057	0.00592
M2- > 1	0	5740	0	4170
M3- > 1	0	8870	0	3850
M1- > 2	0	8330	0	3960
M3- > 2	1510	10000	9800	6230
M1- > 3	0	8450	0	3350
M2- > 3	0	8690	160	4350
ΘMill Creek	0.00000	0.06287	0.00150	0.00992
ΘBeaver Creek	0.00000	0.00160	0.00003	0.00023
ΘMiddle Fk. S. Platte	0.00000	0.03480	0.00210	0.04037
M2- > 1	0	5700	0	1820
M3- > 1	0	5620	0	1280
M1- > 2	0	8260	0	2790
M3- > 2	0	9030	60	4190
M1- > 3	0	6670	0	1490
M2- > 3	0	8470	0	3470
ΘDeer Creek	0.00007	0.07373	0.00243	0.04509
ΘSalina Creek	0.00000	0.04627	0.00030	0.00905
ΘMill Creek Creek	0.00000	0.00753	0.00110	0.00316
M2- > 1	0	9290	0	4660
M3- > 1	0	7430	0	1920
M1- > 2	0	8330	0	3150
M3- > 2	0	8330	0	2300
M1- > 3	0	6350	0	1560
M2- > 3	0	7090	0	1670

#### Historical climate oscillations

Divergence time estimates based on our BEAST analysis suggest that *P. badia* diverged from its sister species *P. regularis* approximately 2.65 million ybp, near the end of the Pliocene Epoch (Fig. 7). The clades forming the initial divergence within *P. badia* (Old Colorado Plateau and Old Rio Grande) dated to 1.59 million ybp, or the middle Pleistocene, with the latest diverging Widespread clade beginning approximately 830,000 ybp. The Widespread subclade containing all localities from Alaska is well supported within the Widespread clade (see Fig. 3) and diverged approximately 110,000 ybp, prior to the last two glacial maxima (Fig. 7).

**Fig. 6:**
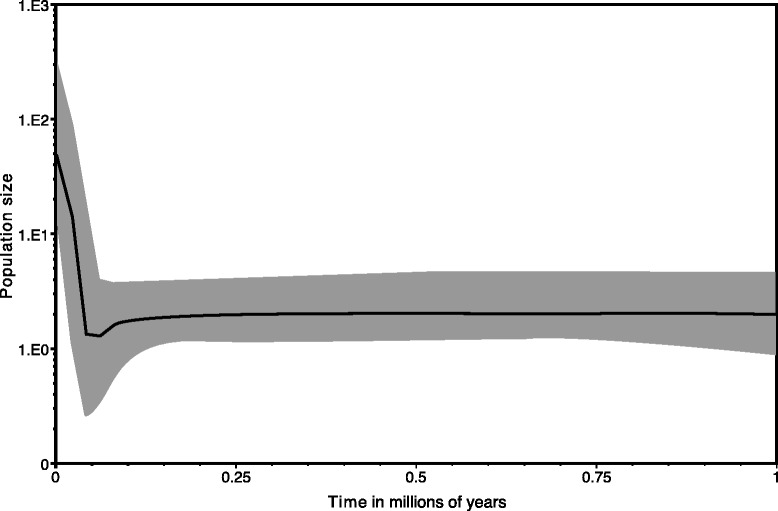
A Bayesian skyline plot showing the recent demographic history of *P. badia,* with time in millions of years ago shown along the x-axis and effective population size shown along the y-axis

The Bayesian Skyline Plot detected a change in effective population size in the late Pleistocene, showing a slight decrease beginning ~100,000 ybp and continuing until ~ 60,000 ybp, followed by a rapid increase from ~60,000 ybp to present (Fig. 6). Fu’s F_S_ was highly negative and statistically significant (*F*_*S*_ = -29.73, *p* < 0.001), also indicative of a rapid demographic expansion. A negative Tajima’s D value further indicated recent expansion; however it was not statistically significant (*D* = −0.486, *p* = 0.10).

## Discussion

### Hydrologic connectivity vs. overland dispersal

Despite having a winged adult stage, some aquatic insects [18] have been shown to exhibit patterns of population structure consistent with a stream hierarchy model [19] in which the majority of genetic variation can be explained by differences between drainage basins. This pattern is expected in aquatic insects that have limited flight ability, or that exhibit high habitat fidelity to stream corridors [4]. Our AMOVA results suggest that *P. badia* does not fall into this category of aquatic insects as only 26.15 % (*p* < 0.001) of genetic variation was explained by differences in drainage basin. This result provides initial evidence that hydrologic connectivity is not a major determinant of genetic structure across broad geographic scales in *P. badia*.

We emphasize that the above stated conclusion, that hydrologic connectivity is not a primary driver in the genetic structure of this species, is scale dependent. Our sampling density allowed us to test for dispersal across tens to hundreds of kilometers. At local levels (tens of kilometers), contemporary dispersal among networks of stream corridors likely results in high levels of connectivity. Larger tracts of connectivity would be expected in regions of the landscape with a greater density of habitats meeting the ecological requirements of *P. badia*; however, connectivity across broad distances within drainages is not necessarily expected. For example, although high order, low-elevation streams provide a hydrologic connection between patches of mid-elevation habitat suitable for *P. badia*, these low elevation connecting rivers may not meet the ecological requirements of the species (due to temperature, gradient, dissolved O_2_, etc.), thereby preventing in-stream dispersal of aquatic forms. Low elevation regions may also be sufficiently expansive as to prevent dispersal of winged adults to opposite slopes of large drainages [4, 20].

Thus, it may have been more biologically realistic to designate *a priori* groups according to sub-drainage. The geographic distribution of our sampling localities, however, did not allow us to designate *a priori* groups according to sub-basin across the study area, while retaining sufficient replicates for statistical analysis of all the groups. While it is possible that grouping according to sub-drainage basin would explain a greater percentage of mtDNA diversity than our AMOVA as presently organized, it is clear from the geographic distribution of the various clades (Fig. 3) that dispersal events for this taxon are not exclusively dependent on hydrologic connections, as the Widespread clade, Northern Rockies clade, and PNW clade all contain haplotypes from two or more major drainage basins.

The presently observed distribution of haplotypes in the Widespread clade suggests that this lineage achieved overland connectivity from the southern Rocky Mountains to Alaska in the relatively recent history of the species, as recently as 100,000 ybp according to our dating analysis. This pattern would only be observed if overland dispersal across drainage boundaries were a dispersal mechanism in the group. The Northern Rockies clade provides what appears to be a recent example of inter-basin transfer with very closely related haplotypes being present in both the upper Columbia (Kootenay River), and Hudson Bay drainages (Bow River, Lee Creek, and Spray River). While post-glacial colonization into the upper Columbia drainage (Kootenay River) could have proceeded from populations residing at lower elevations in the same drainage, *P. badia* in the upper Hudson Bay drainage (Bow River, Lee Creek, and Spray River) either must have colonized by way of overland dispersal in the last ~20,000 years since the whole of the Hudson Bay drainage was glaciated during the Last Glacial Maximum (LGM), or closely tracked the recession of periglacial lakes formed by the melting Laurentide ice sheet. Beyond the Northern Rockies clade however, all other clades except the Western Great Basin (which consists of a single locality) also show closely related haplotypes that span drainage boundaries. The results of our gene flow analysis provide empirical support to the observed patterns of inter-basin movement discussed above.

Gene flow estimates were highest between localities in separate drainage basins in two of the three locality sets. This suggests that in certain areas of the species distribution, dispersal via overland flight across drainage boundaries may be more common than dispersal via overland flight (or larval drift) between distant localities (100–500 km distant) on the same river. While the geographic distribution of the clades and the gene flow analysis show that mtDNA diversity for *P. badia* is not structured exclusively along drainage basin boundaries, as predicted for organisms that are extreme headwater specialists, the headwater model of dispersal [20] appears to be an important mechanism in shaping the population structure of *P. badia*. We expect headwater dispersal to be of particular importance in areas where drainage basin boundaries fall within (or near) the elevational distribution of the species, as observed for the boundary between the Green River and Hoback River localities. Our analysis also found isolation by distance to be a dispersal related mechanism shaping genetic structure. While isolation by distance was non-significant for the Northern Rockies, Old Colorado Plateau, Old Rio Grande, and Pacific Northwest clades, a significant correlation was found in the Widespread clade, which contains over half of the localities sampled in the present study, and has the largest geographical footprint. Finally, although overland dispersal of the species is clearly evident, the very high overall Φ_ST_ of 0.91 (*p* < 0.001) indicates that dispersal events between geographically distinct localities are rare. This conclusion is also consistent with the presence of several well-supported subclades that are comprised of specimens from a single geographic locality, or multiple geographically proximate localities (Fig. 4).

### Dates of divergence

Our dating analysis places the divergence of *P. badia* from its sister species *P. regularis* at the end of the Pliocene approximately 2.5 million ybp. This divergence date is coincident with the of xerification of the Columbia basin associated with the Cascadian orogeny [21], a vicariance event that corresponds with speciation events in many plant and animal species distributed in western North America [22, 23], including stoneflies of the Great Basin [24]. This finding provides evidence that our use of a general insect mtDNA mutation rate (as opposed to a more lineage specific calibration) to calibrate our molecular clock has produced a plausible working hypothesis for divergence dates in *P. badia*. Still, we note that the precision of the dates here discussed should be re-evaluated as more calibration data become available for pteronarcyid stoneflies.

### Historical climatic oscillations

Several studies have shown Pleistocene glacial cycles to be one of the main drivers in shaping genetic structure of various aquatic insect species across Europe [3, 6, 7, 25]. Our data suggest that historical climate oscillations have been an important factor in shaping the current and past distribution of *Pteronarcella badia* as well, and may have given rise to many of the presently observed patterns of genetic structure. The presence of several, highly differentiated mitochondrial lineages confined to discrete geographic regions corroborates evidence for multiple glacial refugia seen in other aquatic groups distributed across glaciated regions [6, 26]. In particular, glacial refugia have likely been key in the differentiation of the Pacific Northwest and Northern Rockies clades, as refugia in the Pacific Northwest and Bitterroot Valley in Montana are well documented in vertebrate and plant groups [22, 23].

**Fig. 7:**
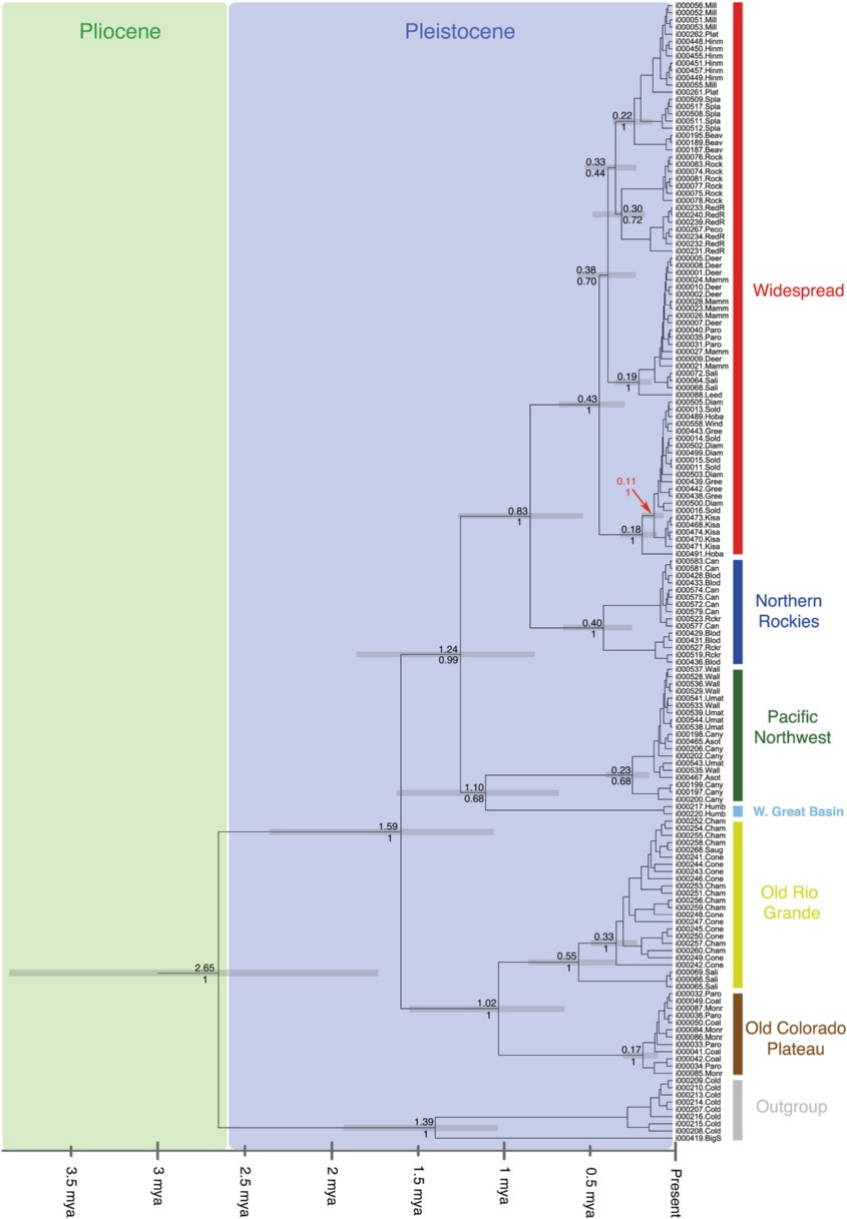
A BI tree reconstruction based on 2518 bp of mtDNA. Pliocene and Pleistocene epochs indicated with colored boxes and major clades are color coded in the same color scheme as Fig. 4. Date of divergence is shown above node lines with posterior probabilities shown below node lines. Error associated with estimated dates is shown by gray rectangles at each dated node. The clade containing the Alaskan haplotypes is indicated by the red arrow

Additional evidence of past glacial cycles affecting present genetic patterns is our unexpected finding that all haplotypes from Alaskan localities occur as a subclade within the Widespread clade, despite their being geographically closer to the Northern Rockies and Pacific Northwest clades (Fig. 2). Our divergence dating analysis estimated that the clade of Alaskan haplotypes diverged from the remaining members of the Widespread clade from 100,000 to 200,000 ybp (Fig. 7), suggesting at least intermittent connectivity of the Widespread clade existed between Alaska and the lower latitudes of the species distribution through the Pleistocene. We hypothesize that connectivity of the Widespread clade was interrupted during the most recent glacial periods, and certainly the LGM, as the Cordilleran Ice Sheet pushed *P. badia* into northern (Alaskan) refugia [27, 28] and southern refugia. As the continental ice sheets retreated northward, our results show that post-glacial re-colonization expanded from refugial Montana localities [9, 22] and not localities containing Widespread clade haplotypes that were previously connected with the Alaskan subclade, as evidenced by all haplotypes from the southern Canadian sample localities falling in the Northern Rockies clade.

This raises the question: Is there presently connectivity between the Alaskan and southern groups? No evidence of present genetic connectivity exists. The Canadian sample localities that are closest geographically to Alaska contain haplotypes of the genetically distant Northern Rockies clade (Figs. 3 and 4). Further, we found no confirmed records of *P. badia* in northern British Columbia and the Yukon through extensive personal contacts with aquatic biologists in southeastern Alaska, British Columbia, Yukon territories, or through a careful search of Canadian stonefly checklists [29, 30] and the GBIF database. We, therefore, conclude that it is likely that post-glacial expansion has not yet resulted in connectivity between the northern Widespread subclade and southern refugial groups (Northern Rockies, Pacific Northwest, or Widespread clades), resulting in an actual gap in the present species distribution.

Although the presence of the Cordilleran and Laurentide ice sheets would have caused the distribution of *P. badia* to contract northward into the Bering refugia, or into refugia south of the glacial ice sheets, in general our analysis of past population demographics with Fu’s F_s_ and Bayesian skyline plot data (Fig. 6) show evidence of recent demographic expansion roughly coincident with the LGM, a pattern that has been observed in other North American and European aquatic insect groups [6, 24]. We hypothesize that the rapid increase in effective population size in the last 60,000 years shown by our Bayesian skyline plot is driven by fluctuating climate associated with the LGM. Cooling global temperatures preceding the LGM would have likely resulted in southward expansion into lower latitudes. In addition to latitudinal shifts, distributions would have also shifted to lower elevations within un-glaciated drainages. This could result in longer tracts of habitat connectivity and *P. badia* being able to inhabit higher order streams and rivers than would be possible during interglacial periods. Our sampling of haplotypes from the Colorado Plateau and southern Rockies indeed show patterns consistent with a recent southward range expansion. The earliest diverging clades in our topology (Old Colorado Plateau and Old Rio Grande) occur in the southern most limits of the present species distribution. According to our divergence dating, these lineages in the southern Great Basin and Rio Grande drainages began differentiating in the early-middle Pleistocene ~1.5 million ybp. However, haplotypes from the most recently diverged Widespread clade are also abundant in the southern limits of the species distribution; and in three instances occur at the same localities as Old Colorado Plateau and Old Rio Grande haplotypes (Coal Creek, Parowan Creek, and Salina Creek). This observation is consistent with recent southward expansion of the Widespread clade resulting in secondary contact with the Old Colorado Plateau and Old Rio Grande lineages.

Finally, while cooling patterns would have allowed for southern expansion, the retreat of the continental ice sheet following the LGM has resulted in a subsequent expansion of the Northern Rockies clade into formerly glaciated latitudes in Canada. Northern, post-LGM expansion of the Old Colorado Plateau and Old Rio Grande lineages is also an alternative explanation for the pattern of secondary contact between those clades and the Widespread clade described in the previous paragraph. Thus, both warming and cooling cycles associated with recent glacial maxima are likely drivers in the recent range expansion of the group.

### Presence of cryptic lineages

While our mitochondrial data reveal the presence of several highly divergent clades (3–5 % pairwise distance), the nuclear locus only shows distinctiveness in the Pacific Northwest and Western Great Basin clades (Fig. 2). This is interesting considering that the Old Colorado Plateau and Old Rio Grande mitochondrial clades showed earlier divergence dates, but lack variation at the nuclear locus. While this is unexpected, it is possible that the Old Colorado Plateau and Old Rio Grande clades have simply not yet accumulated changes at the locus we examined, but would show nuclear variation if other loci were examined. This lack of fixed variation could be due to having a larger effective population size than the Pacific Northwest lineage, and thus slower lineage sorting. An alternative explanation is that sufficient gene flow with other clades has occurred to degrade nuclear variation, but migrant mitochondrial haplotypes are sufficiently rare as to not be detected by our sampling. In contrast, 35 of the 36 individuals sampled from the Pacific Northwest and Western Great Basin sample localities showed distinctiveness in at least one of six nucleotide positions in 28S, with many individuals showing substitutions at all six positions (outgroup taxa had fixed variation in at least eight positions in 28S, all different than the variable sites in the ingroup) (Fig. 2). Since we did not detect any haplotypes from the other five clades in the Pacific Northwest and Western Great Basin clades, we suspect that the lack of fixation of these six sites in 28S is due to insufficient time passage to allow the fixation rather than a result of secondary contact with individuals from other clades. The lack of haplotypes from other clades being present in the Pacific Northwest also provides evidence that the Pacific Northwest clade remained in isolation through much of the Pleistocene, despite the range expansions and contractions, which resulted in secondary contact between the Old Colorado Plateau, Old Rio Grande and Widespread clades. Additional analysis of morphological characters and molecular data from Pacific Northwest specimens is currently underway to explore the potential presence of a new candidate species. Finally, our finding that the Pacific Northwest is a phylogeographically important region for *P. badia* is consistent with patterns seen in other western North American taxa [23, 31].

## Conclusion

Our phylogeographic analysis of *P. badia* reveals a complex history of isolation and multiple invasions among some areas and a cryptic lineage in the Pacific Northwest. The study provides evidence of multiple glacial refugia and suggests that historical climatic oscillations have been important mechanisms in determining genetic structure of insects in western North America. Our ability to generate a large mitochondrial data set through mitochondrial genome reconstruction greatly improved nodal support of our mitochondrial gene tree and allowed us to make stronger inference of relationships between lineages and timing of divergence events. We emphasize that due to the limited signal in the nuclear locus we examined, our findings are based almost entirely on mitochondrial data. As such, many of our findings will need to be re-evaluated when additional, informative, nuclear loci become available. This work adds to the ever-growing list of studies that highlight the potential of next-generation DNA sequencing in a phylogenetic context to improve molecular data sets in understudied groups.

## Methods

### Sampling and tissue preparation

We determined the approximate range of *P. badia* through species checklists [29, 30] online databases [32], personal communications with stonefly experts, and museum records. We collected *P. badia* nymphs and adults from 30 localities throughout its known distribution in the western United States. Where densities were sufficiently high, we collected at least ten individuals per locality. Samples for seven additional localities in Canada and Alaska were sent to us by remote collectors or obtained from the stonefly collection at the Monte L. Bean Life Science Museum at Brigham Young University. Although the species is known to occur in both Alaska and southern regions of Canada, we were unable to find confirmed localities for the species in northern British Columbia, northern Alberta or the Yukon, indicating an apparent gap in the known species distribution.

For outgroup sampling, we obtained up to ten individuals from two localities of the sister species *P. regularis* from both personal collecting efforts and the Monte L. Bean Life Science Museum. All samples collected through personal efforts were preserved in 100 % EtOH and stored at −80 °C until DNA could be extracted. Ten individuals per locality were included in the phylogenetic analysis. We determined that 10 individuals per locality would adequately sample haplotypes based on a pilot study data, which showed low haplotypic diversity within localities but high haplotypic diversity between localities. For localities where fewer than ten individuals were obtained, we used all available individuals for that locality. DNA was dissected from leg or thoracic muscle tissue and extracted using the Qiagen® DNeasy™ protocol. All DNA sequences were uploaded to GenBank as accession numbers KU180827 through KU182359. Collection information is summarized in Table 2 and deposited in the Dryad Digital Repository (doi:10.5061/dryad.c1sd4) . GenBank accession numbers are listed by gene in Additional file 1: Table S1.

**Table 2:** Sample locality data

River/Stream name	Code	N=	St./Prov	County	Country	Lat	Lon	BYU code
Clearwater River	~	1	AB	Clearwater	Canada	51.9899°	−115.3913°	563
Lee Creek	~	1	AB	Cardston	Canada	~	~	577
Spray River	~	1	AB	Banff Park	Canada	~	~	565
Crooked Creek	~	5	AK	Kenai Penn.	USA	60.5642°	−149.9545°	549–553
Kisaralik River	Kisa	8	AK	Bethel	USA	~	~	468–475
*Big Springs	BigS	1	CA	Siskiyou	USA	~	~	419
*Cold Stream (Creek)	Cold	5	CA	Sierra	USA	39.5239°	−120.2884°	207–211
Thomas Creek	~	1	CA	Modoc	USA	~	~	564
Beaver Creek	Beav	10	CO	Gunnison	USA	38.4947°	−107.0319°	187–196
Conejos River	Cone	10	CO	Conejos	USA	37.0574°	−106.1903°	241–250
Hinman Creek	Hinm	10	CO	Routt	USA	40.7512°	−106.8383°	448–457
La Plata River	Plat	6	CO	La Plata	USA	37.2752°	−108.0334°	261–266
Middle Fk S.Platte R.	Spla	10	CO	Park	USA	39.2189°	−105.9941°	508–517
Saugache Creek	Saug	1	CO	Saguache	USA	38.1295°	−106.4568°	268
Rock Creek	Rock	10	ID	Cassia	USA	42.3350°	−114.2822°	074–83
Blodgett Creek	Blod	10	MT	Ravalli	USA	46.2891°	−114.1609°	428–437
Rock Creek	Rckr	10	MT	Granite	USA	46.7109°	−113.6736°	518–527
Chama River	Cham	10	NM	Rio Arriba	USA	36.9124°	−106.5732°	251–260
Pecos River	Peco	1	NM	San Miguel	USA	35.6934°	−105.6942°	267
Red River	RedR	10	NM	Taos	USA	36.6983°	−105.4764°	231–240
N. Fk Humboldt River	Humb	4	NV	Elko	USA	41.5783°	−115.9309°	217–220
Canyon Creek	Cany	10	OR	Grant	USA	44.2913°	−118.9570°	197–206
Umatilla River	Umat	9	OR	Umatilla	USA	45.6745°	−118.7587°	538–547 less 42
Walla Walla River	Wall	10	OR	Umatilla	USA	45.9214°	−118.3737°	528–537
Coal Creek	Coal	8	UT	Iron	USA	37.6717°	−113.0413°	041–50 less 44,48
Deer Creek	Deer	9	UT	Garfield	USA	38.0125°	−111.9740°	001–10 less 6
Diamond Fork River	Diam	10	UT	Utah	USA	40.0787°	−111.3708°	498–507
Leeds Creek	Leed	1	UT	Washington	USA	37.2655°	−113.3688°	88
Mammoth Creek	Mamm	10	UT	Garfield	USA	37.6280°	−112.4567°	021–30
Mill Creek	Mill	13	UT	San Juan	USA	38.4836°	−109.4086°	051–63
Monroe Creek	Monr	4	UT	Sevier	USA	38.6105°	−112.1058°	084–87
Parowan Creek	Paro	10	UT	Iron	USA	37.8085°	−112.8094°	031–40
Salina Creek	Sali	10	UT	Sevier	USA	38.8791°	−111.5531°	064–73
Soldier Creek	Sold	10	UT	Utah	USA	39.9647°	−111.3098°	011–20
Asotin Creek	Asot	2	WA	Asotin	USA	46.3317°	−117.0828°	465,467
Green River	Gree	9	WY	Sublette	USA	43.0181°	−110.1182°	438–447 less 445
Hoback River	Hoba	5	WY	Sublette	USA	43.2455°	−110.4766°	487–491
Wind River	Wind	1	WY	Fremont	USA	43.5254°	−109.6170°	558

* Indicates sample localities of outgroup taxa. "Code" is the stream name abbreviation appended to the specimen names in Fig. 4. "Code" is the locality code which is appended to the seqeunce name in Figs. 4 and 7. "BYU code" indicates the last three digits of the BYU voucher number is the specimen number used in Figs. 4 and 7 (the first three digits are "i000" for all samples)

### Mitochondrial genome reconstruction

#### Pyrosequencing, assembly, and annotation

We pooled whole genomic DNA extracted from a single *P. badia* individual collected on the Diamond Fork River (Table 2) with DNA from three other taxa on a 454 half plate. Library preparation and sequencing were performed on a 454 Life Sciences Genome Sequencer FLX at the Brigham Young University DNA Sequencing Center. Following pyrosequencing, reads were assembled using Newbler v.2.6 (454 Life Sciences 2006–2011). We identified mitochondrial DNA contigs using Basic Local Alignment Search Tool (BLAST), and assembled the mitochondrial contigs using the closely related (sister genus) *Pteronarcys princeps* (Hagen) mt genome [14] as a reference. To fill in small gaps between the *P. badia* contigs, we designed PCR primers flanking gap regions and amplified the gaps using DNA extracted from the same individual that was sequenced via 454 pyrosequencing. To amplify part of the A + T rich control region not recovered in the Newbler assembly, we used Phusion High-fidelity DNA Polymerase (New England BioLabs, Ipswich, MA) on an Eppendorf Mastercycler® pro (Hamburg, Germany) in a 12.5 μL reactions containing 3 μL of DNA template, 2.25 μL nuclease free water, 0.5 μL dNTP’s, 0.5 μL each primer, and 6.25 μL Phusion polymerase using the following thermal profile: 2 min. at 95 °C, followed by 35 cycles of 30 s @ 95 °C, 30 s annealing @ 50 °C, and 2 min. extension @ 72 °C, with a final elongation step of 4 min at 72 °C. We used MOSAS [33] to identify protein coding, ribosomal RNA, and tRNAscan-SE v1.21 [34] as implemented in MOSAS, as well as alignment to other insect mt genomes to identify t-RNA regions. We identified open reading frames and annotated the genome in Geneious v5.5.5 [35]. The complete genome was deposited in GenBank with accession number KU182360.

### Phylogenetic analysis and hypothesis testing

#### PCR amplification and sequencing

We targeted portions of five, mitochondrial protein coding genes for PCR amplification: ATP6 (506 bp), COI (844 bp), COIII (711 bp), CYTB (795 bp), and ND6 (493 bp); as well as a portion of nuclear ribosomal 28S (~1,050 bp) for 275 individuals (265 ingroup, ten outgroup) from 40 sample localities (38 ingroup, two outgroup) across western North America. Genes from the mitochondrial locus were amplified via PCR using primer pairs designed from the annotated mt genome, which we generated through 454 pyrosequencing. We chose primer locations flanking regions that showed moderate to high variation in an alignment between the mt genomes of *P. badia* and the closely related *P. princeps* in an attempt to select markers that would give maximum resolution to our species-level data set. We chose 28S as the second locus because it showed variation at six positions in a pilot study that considered a sub-sample of individuals from geographically distant localities. All primers used for data set generation are listed in Table 3.

**Table 3:** A list of primers used in the analysis. See text for primer sources

Genes	Primer name	Sequence (5′-3′)	Length	Direction	Frag. size
COI	Ptero.COIAF	ACTTGGCCAACCTGGTTCTCTT	22	Forward	844
	Ptero.COIBR	GTGGAGGGTTGCTAGTCAGCTA	22	Reverse	
ATP6, COIII	Ptero.ATP6BF	CACAGGACACGCTGGTAGAACT	22	Forward	1216
	Ptero.COIIIR	AGTGTCAATATCAGGCTGCTGCT	23	Reverse	
ND6, CYTb	Ptero.ND6BF	CCCAAATAAGTCACCCCTTAGCCA	24	Forward	1288
	Ptero.CYTBR	CATTCTGGTTGAATGTGGACGGG	23	Reverse	
28S	Ptero.28SBF	ACACGTTGGGACCCGAAAGA	20	Forward	880
	Ptero.28SBR	TTCCAGGGAACTCGAACGCTT	21	Reverse	

We performed PCR with a Peltier PTC-225 DNA Engine Tetrad Thermal Cycler (MJ Research, Inc., Waltham, MA) in 12.5 μL reactions containing 3 μL of DNA template, 2.25 μL sterile distilled water, 0.5 μL each primer, and 6.25 μL GoTaq mastermix using the following thermal profile: 2 min. at 95 °C, followed by 35 cycles of 30 s @ 95 °C, 30 s annealing @ 50 °C, and 2 min. extension @ 72 °C, with a final elongation step of 4 min at 72 °C. We verified successful amplification using ultraviolet visualization following gel electrophoresis with a 1 % agarose gel. We purified PCR product with Millipore MultiScreen_μ96_ filter plates. (Bio101, Inc., Vista, CA). The purified DNA was sequenced in 10.5 μL reactions using the ABI Big Dye terminator standard protocol (Applied Biosystems, Inc., Palo Alto, CA) and sequenced at the BYU DNA Sequencing Center using an ABI 3730XL automated sequencer (Applied Biosystems). We edited DNA sequences using Geneious v5.5.5 and aligned using MAFFT v6 [36]. We aligned in MAFFT for its ability to consider secondary RNA structure [37] and its use of an iterative process to quickly obtain optimal alignments. Protein coding genes were aligned with the G-INS-i algorithm with the scoring matrix set to 1PAM/k = 2, gap penalty = 1.53, and offset value = 0.25. For ribosomal RNA genes, the Q-INS-i algorithm (which considers secondary RNA structure) was used with the scoring matrix set to 1PAM/k = 2, gap penalty = 1.53, and offset value = 0.1. We translated alignments into amino acid sequences in Geneious v5.5.5 to detect unexpected stop codons, which can indicate the presence of nuclear pseudogenes [38]. We also scanned for the presence of ambiguous bases in chromatograms as additional evidence of pseudogene contaminants [39, 40]. Because mtDNA is inherited as a single unit, we trimmed the ends of each mitochondrial alignment and concatenated alignments into a single file using Mesquite v2.75 [41].

#### What is the population structure?

We calculated genetic diversity, and haplotype and nucleotide diversities in DnaSP v9. To estimate overall population structure, we calculated Φ_ST_ values in Arlequin v3.5 [42]. We used TCS v1.21 [43] to generate haplotype networks for both loci in order to visualize the geographic distribution of haplotypes, and to determine if the data fit the assumption of being “tree-like”.

We performed tree reconstruction using a Maximum Likelihood (ML) approach in RAxML v7.2.6 [44]. After eliminating redundant haplotypes/genotypes, the data set was partitioned by locus, and run with the GTR + I + G model of evolution for 100 replicates followed by 500 bootstrap replicates using the rapid bootstrap algorithm [45]. We identified appropriate models of evolution for implementation in ML analysis, and all subsequent approaches with jModelTest v0.1.1 [46] under the Akaike information criterion [47].

#### Importance of hydrologic connectivity vs. overland dispersal

To test the importance of hydrologic connectivity in determining population structure, we performed AMOVA in Arlequin v3.5 [48] and defined *a priori* groups according to major drainage basin as follows: Columbia, Colorado River, Great Basin, Rio Grande, Yukon, and Hudson Bay, and Kenai. We assumed for our null hypothesis that if hydrologic connectivity (as a mechanism for dispersal) is important in determining population structure, as it is with many freshwater obligates such as fish, the AMOVA should show that a large percentage of the mtDNA diversity is explained by differences among drainage basin [17, 48–50]. To further test the importance of waterway connections, we performed divergence dating in BEAST v1.6.1 [51] to determine whether divergence from the outgroup (sister species) or between lineages was coincident with known river transfer events in North America [52]. We used the relative rate test [53] implemented in MEGA v5.04 [54] to test whether our data fit the assumptions of a molecular clock. We calibrated the clock using a mean mutation rate of 3.5 % per lineage per million years, an estimated rate for protein-coding insect mitochondrial DNA [55]. We used a generalized rate in the absence of appropriate fossil data, or a well-calibrated molecular phylogeny of stoneflies. To account for uncertainty in the actual mtDNA mutation rate, we specified a standard deviation of 20 % of the mean rate. We specified a relaxed uncorrelated lognormal clock and ran two chains of 100 million generations each, sampling every 2,000 generations with a GTR + gamma + I model of evolution. Based on visualization of the tracings in the program Tracer v1.5 [51], we discarded the first 10 % of the trees as burn-in. We combined results from each run using LogCombiner v1.6.1 [51].

We compared the importance of overland flight to dispersal through water connectivity by comparing gene flow estimates between localities with and without water connections. We selected localities to include in the gene flow analysis based on three criteria: having geographic proximity and direct hydrologic connectivity to an intra-basin locality, geographic proximity to an inter-basin locality (thus, not connected hydrologically), and having several sample replicates (at least *n* = 5). Thus, we estimated gene flow between three localities, two having a direct hydrologic connection between them, and a third that lies in an adjacent drainage basin. This analysis pattern was repeated three times across our sampled area. One locality in the analysis, Salina Creek, had individuals from multiple clades (Old Rio Grande and Widespread). We removed the specimens from the Old Rio Grande clade prior to analysis such that gene flow was only estimated between specimens belonging to the Widespread clade. Gene flow estimates were calculated using MIGRATE-n v3.2.7 [56, 57], chosen for its implementation of coalescent-based methods in a Bayesian framework. We preferred a Bayesian approach based on simulation studies that show it to be more straightforward than Maximum Likelihood methods for estimating gene flow using a single locus [57] (the nuclear locus was monomorphic for all samples included in the gene flow analysis). Following experimental runs of varying lengths to test for convergence, final analyses consisted of a single long chain sampled for 5 million generations (sampling increment = 100 generations, burnin = 100,000 trees), independently replicated three times.

To examine isolation by distance as a mechanism for determining population structure, we searched for a correlation between Slatkin’s [58] linearized *F*_*ST*_ versus log (geographic distance) in each of the major clades, using a Mantel *T*-test implemented in IBDWS v3.23 [59]. The clade containing samples from Alaska was analyzed with, and without Alaskan samples to determine their impact as potential outliers based on their disjunct geographic location.

#### Importance of climatic oscillations

To test the effect of past climate oscillations on historical demography, we estimated changes in effective population size through time using a Bayesian skyline plot [60] implemented in BEAST v1.6.1 [51]. We removed outgroup taxa and ran two chains of 80 million generations under a relaxed lognormal clock prior and a mutation rate of 3.5 % per lineage per million years and standard deviation of 20 % of the mean. We combined multiple runs in LogCombiner v1.6.1 and visualized tracing, confirmed convergence across multiple runs, and generated the skyline plot using Tracer v1.5 [51]. To further explore the impact of known climatic events on species demography, we mapped major climate transitions (Pliocene onset, Pleistocene onset, last glacial maximum) onto the tree generated in our divergence dating analysis (described above). As an additional test for recent patterns in population dynamics, we calculated Tajima’s D [53] in DnaSP v5 [61] and Fu’s F in Arlequin v3.5 [42].

## Availability of supporting data

- The data set supporting the results of this article are available in GenBank (KU180827-KU182359 and KU182360) and the Dryad repository (doi:10.5061/dryad.c1sd4) [62].

### Ethics

All field-work was conducted in compliance with local legislation. All specimens were collected on public lands for which special permits were not required. No endangered or regulated invertebrates were affected by this study.

## Additional file

Additional file 1: Table S1. GenBank accession numbers for 28S and mitochondrial genes included in the study. (DOCX 39 kb)
