# Supplementary material for: Climate oscillations, glacial refugia, and dispersal ability: factors influencing the genetic structure of the least salmonfly, Pteronarcella badia (Plecoptera), in Western North America
Source: BMC Evol Biol. 2015 Dec 12;15:279. doi: 10.1186/s12862-015-0553-4 (PMC4676849; doi:10.1186/s12862-015-0553-4)
Supplement: Additional file 1: Table S1. — GenBank accession numbers for 28S and mitochondrial genes included in the study. (DOCX 39 kb) [file 12862_2015_553_MOESM1_ESM.docx]

**Table S1 GenBank accession numbers for 28S and mitochondrial genes included in the study**

| **River/Stream Name** | **BYU code** | **28S** | **ATP6** | **COI** | **COIII** | **CYTB** | **ND6** |
| --- | --- | --- | --- | --- | --- | --- | --- |
| Clearwater River | 563 | ~ | ~ | KU181580 | ~ | KU182091 | KU182351 |
| Lee Creek | 577 | ~ | ~ | KU181583 | ~ | KU182094 | KU182354 |
| Spray River | 565 | ~ | ~ | KU181582 | ~ | KU182093 | KU182353 |
| Crooked Creek | 549-553 | ~ | ~ | KU181585-KU181579 | ~ | KU182096-KU182090 | KU182356-KU182350 |
| Kisaralik River | 468-475 | KU181015-KU181022 | KU181266-KU181273 | KU181517-KU181524 | KU181777-KU181784 | KU182028-KU182035 | KU182288-KU182295 |
| *Big Springs | 419 | KU180983 | KU181234 | KU181485 | KU181745 | KU181996 | KU182256 |
| *Cold Stream (Creek) | 207-211 | KU180932-KU180940 | KU181183-KU181191 | KU181434-KU181442 | KU181694-KU181702 | KU181945-KU181953 | KU182205-KU182213 |
| Thomas Creek | 564 | ~ | ~ | KU181581 | ~ | KU182092 | KU182352 |
| Beaver Creek | 187-196 | KU180912-KU180921 | KU181163-KU181172 | KU181414-KU181423 | KU181674-KU181683 | KU181925-KU181934 | KU182185-KU182194 |
| Conejos River | 241-250 | KU180955-KU180964 | KU181206-KU181215 | KU181457-KU181466 | KU181717-KU181726 | KU181968-KU181977 | KU182228-KU182237 |
| Hinman Creek | 448-457 | KU181003-KU181012 | KU181254-KU181263 | KU181505-KU181514 | KU181765-KU181774 | KU182016-KU182025 | KU182276-KU182285 |
| La Plata River | 261-266 | KU180975-KU180980 | KU181226-KU181231 | KU181477-KU181482 | KU181737-KU181742 | KU181988-KU181993 | KU182248-KU182253 |
| Middle Fk S.Platte R. | 508-517 | KU181038-KU181047 | KU181289-KU181298 | KU181540-KU181549 | KU181800-KU181809 | KU182051-KU182060 | KU182311-KU182320 |
| Saugache Creek | 268 | KU180982 | KU181233 | KU181484 | KU181744 | KU181995 | KU182255 |
| Rock Creek | 074-83 | KU180897-KU180906 | KU181148-KU181157 | KU181399-KU181408 | KU181659-KU181668 | KU181910-KU181919 | KU182170-KU182179 |
| Blodgett Creek | 428-437 | KU180984-KU180993 | KU181235-KU181244 | KU181486-KU181495 | KU181746-KU181755 | KU181997-KU182006 | KU182257-KU182266 |
| Rock Creek | 518-527 | KU181048-KU181057 | KU181299-KU181308 | KU181550-KU181559 | KU181810-KU181819 | KU182061-KU182070 | KU182321-KU182330 |
| Chama River | 251-260 | KU180965-KU180974 | KU181216-KU181225 | KU181467-KU181476 | KU181727-KU181736 | KU181978-KU181987 | KU182238-KU182247 |
| Pecos River | 267 | KU180981 | KU181232 | KU181483 | KU181743 | KU181994 | KU182254 |
| Red River | 231-240 | KU180945-KU180954 | KU181196-KU181205 | KU181447-KU181456 | KU181707-KU181716 | KU181958-KU181967 | KU182218-KU182227 |
| N. Fk Humboldt River | 217-220 | KU180941-KU180944 | KU181192-KU181195 | KU181443-KU181446 | KU181703-KU181706 | KU181954-KU181957 | KU182214-KU182217 |
| Canyon Creek | 197-206 | KU180922-KU180931 | KU181173-KU181182 | KU181424-KU181433 | KU181684-KU181693 | KU181935-KU181944 | KU182195-KU182204 |
| Umatilla River | 538-547 less 42 | KU181068-KU181076 | KU181319-KU181327 | KU181570-KU181578 | KU181830-KU181838 | KU182081-KU182089 | KU182341-KU182349 |
| Walla Walla River | 528-537 | KU181058-KU181067 | KU181309-KU181318 | KU181560-KU181569 | KU181820-KU181829 | KU182071-KU182080 | KU182331-KU182340 |
| Coal Creek | 041-50 less 44,48 | KU180866-KU180873 | KU181117-KU181124 | KU181368-KU181375 | KU181628-KU181635 | KU181879-KU181886 | KU182139-KU182146 |
| Deer Creek | 001-10 less 6 | KU180827-KU180835 | KU181078-KU181086 | KU181329-KU181337 | KU181589-KU181597 | KU181840-KU181848 | KU182100-KU182108 |
| Diamond Fork River | 498-507 | KU181028-KU181037 | KU181279-KU181288 | KU181530-KU181539 | KU181790-KU181799 | KU182041-KU182050 | KU182301-KU182310 |
| Leeds Creek | 88 | KU180911 | KU181162 | KU181413 | KU181673 | KU181924 | KU182184 |
| Mammoth Creek | 021-30 | KU180846-KU180855 | KU181097-KU181106 | KU181348-KU181357 | KU181608-KU181617 | KU181859-KU181868 | KU182119-KU182128 |
| Mill Creek | 051-63 | KU180874-KU180886 | KU181125-KU181137 | KU181376-KU181388 | KU181636-KU181648 | KU181887-KU181899 | KU182147-KU182159 |
| Monroe Creek | 084-87 | KU180907-KU180910 | KU181158-KU181161 | KU181409-KU181412 | KU181669-KU181672 | KU181920-KU181923 | KU182180-KU182183 |
| Parowan Creek | 031-40 | KU180856-KU180865 | KU181107-KU181116 | KU181358-KU181367 | KU181618-KU181627 | KU181869-KU181878 | KU182129-KU182138 |
| Salina Creek | 064-73 | KU180887-KU180896 | KU181138-KU181147 | KU181389-KU181398 | KU181649-KU181658 | KU181900-KU181909 | KU182160-KU182169 |
| Soldier Creek | 011-20 | KU180836-KU180845 | KU181087-KU181096 | KU181338-KU181347 | KU181598-KU181607 | KU181849-KU181858 | KU182109-KU182118 |
| Asotin Creek | 465,467 | KU181013-KU181014 | KU181264-KU181265 | KU181515-KU181516 | KU181775-KU181776 | KU182026-KU182027 | KU182286-KU182287 |
| Green River | 438-447 less 445 | KU180994-KU181002 | KU181245-KU181253 | KU181496-KU181504 | KU181756-KU181764 | KU182007-KU182015 | KU182267-KU182275 |
| Hoback River | 487-491 | KU181023-KU181027 | KU181274-KU181278 | KU181525-KU181529 | KU181785-KU181789 | KU182036-KU182040 | KU182296-KU182300 |
| Wind River | 558 | KU181077 | KU181328 | KU181584 | KU181839 | KU182095 | KU182355 |
